# R31C *GNRH1* Mutation and Congenital Hypogonadotropic Hypogonadism

**DOI:** 10.1371/journal.pone.0069616

**Published:** 2013-07-25

**Authors:** Luigi Maione, Frederique Albarel, Philippe Bouchard, Megan Gallant, Colleen A. Flanagan, Regis Bobe, Joelle Cohen-Tannoudji, Rosario Pivonello, Annamaria Colao, Thierry Brue, Robert P. Millar, Marc Lombes, Jacques Young, Anne Guiochon-Mantel, Jerome Bouligand

**Affiliations:** 1 Université Paris-Sud, Faculté de Médecine Paris-Sud Unité mixte de Recherche en Santé 693, Le Kremlin Bicetre, France; 2 Service d'Endocrinologie et des Maladies de la Reproduction, Hopital Bicetre, Assistance Publique Hopitaux de Paris, Le Kremlin-Bicêtre, France; 3 Département d'Endocrinologie et Centre de Référence des Maladies Rares d'Origine Hypophysaire, Hopital de la Timone, Marseille, France; 4 Service d'Endocrinologie, diabétologie et endocrinologie de la reproduction, Hopital Saint-Antoine, Assistance Publique-Hopitaux de Paris, Paris, France; 5 University of Cape Town Medical School, Medical Research Council, Receptor Biology Research Unit, Institute of Infectious Diseases and Molecular Medicine, Observatory, Cape Town, South Africa; 6 School of Physiology, University of the Witwatersrand Faculty of Health Sciences, Parktown, Johannesburg, South Africa; 7 Université Paris-Sud, Unité mixte de Recherche en Santé 770, Le Kremlin-Bicetre, France; 8 Equipe Physiologie de l'Axe Gonadotrope, Unité de Biologie Fonctionnelle et Adaptative, Sorbonne Paris Cité, Université Paris Diderot-Paris 7, Paris, France; 9 Università degli Studi di Napoli Federico II, Dipartimento di Medicina Clinica e Chirurgia, Sezione di Endocrinologia e Metabolismo, Napoli, Italy; 10 Mammal Research Institute, Faculty of Natural and Agricultural Sciences, University of Pretoria, Pretoria, South Africa and Centre for Integrative Physiology, College of Medicine and Veterinary Medicine, University of Edinburgh, Edinburgh, Scotland; 11 Laboratoire de Génétique moléculaire, Pharmacogénétique et Hormonologie, Hopital Bicetre, Assistance Publique Hopitaux de Paris, Le Kremlin-Bicetre, France; John Hopkins University School of Medicine, United States of America

## Abstract

Normosmic congenital hypogonadotropic hypogonadism (nCHH) is a rare reproductive disease leading to lack of puberty and infertility. Loss-of-function mutations of *GNRH1* gene are a very rare cause of autosomal recessive nCHH. R31C *GNRH1* is the only missense mutation that affects the conserved GnRH decapeptide sequence. This mutation was identified in a CpG islet in nine nCHH subjects from four unrelated families, giving evidence for a putative “hot spot”. Interestingly, all the nCHH patients carry this mutation in heterozygosis that strikingly contrasts with the recessive inheritance associated with frame shift and non-sense mutations. Therefore, after exclusion of a second genetic event, a comprehensive functional characterization of the mutant R31C GnRH was undertaken. Using different cellular models, we clearly demonstrate a dramatic reduction of the mutant decapeptide capacity to bind GnRH-receptor, to activate MAPK pathway and to trigger inositol phosphate accumulation and intracellular calcium mobilization. In addition it is less able than wild type to induce *lh*-*beta* transcription and LH secretion in gonadotrope cells. Finally, the absence of a negative dominance *in vitro* offers a unique opportunity to discuss the complex *in vivo* patho-physiology of this form of nCHH.

## Introduction

The gonadotropin-releasing hormone (GnRH) is essential in mammalian reproduction. This decapeptide, released from hypothalamic GnRH-neurons, triggers an intracellular cascade involving IP3 accumulation, calcium mobilization and MAPK phosphorylation through its cognate receptor GnRHR. The activation of these signaling pathways ultimately stimulates the synthesis and secretion of gonadotropins (LH and FSH) by pituitary gonadotrope cells.

The decapeptide sequence is conserved among most mammals and the amino and carboxyl termini are conserved in mammals and invertebrates [1]–[4].

Mutations of the human *GNRH1* gene, encoding a 92 amino-acid pre-pro-GnRH, are a very rare cause of normosmic congenital hypogonadotropic hypogonadism (nCHH). A frame shift resulting in a failure to translate the GnRH peptide sequence gives rise to nCHH with autosomal recessive inheritance [5]. Subsequently a p.R31C *GNRH1* mutation in which arginine is substituted by cysteine has been described [6], [7]. This is the sole mutation affecting the GnRH decapeptide sequence. The arginine in position 8 of the GnRH decapeptide has been shown to be crucial for biological activity [2]–[4] and shown to interact with an acidic residue in the mouse [8] and in the human [9] GnRH-Rs. This mutation, though identified in two nCHH families in two independent series [6], [7], has not been characterized. In these families, the somehow afflicted individuals were heterozygous. This observation is surprising as the frame shift *GNRH1* mutation only resulted in nCHH in homozygous patients [5].

Here we report on the identification of p.R31C mutation in three individuals in two additional unrelated nCHH families. All the individuals are heterozygous for the mutation. We have undertaken a comprehensive molecular characterization of the mutation in order to understand the mechanism of nCHH in these individuals.

## Results

### Genetic analysis

**Figure 1:**
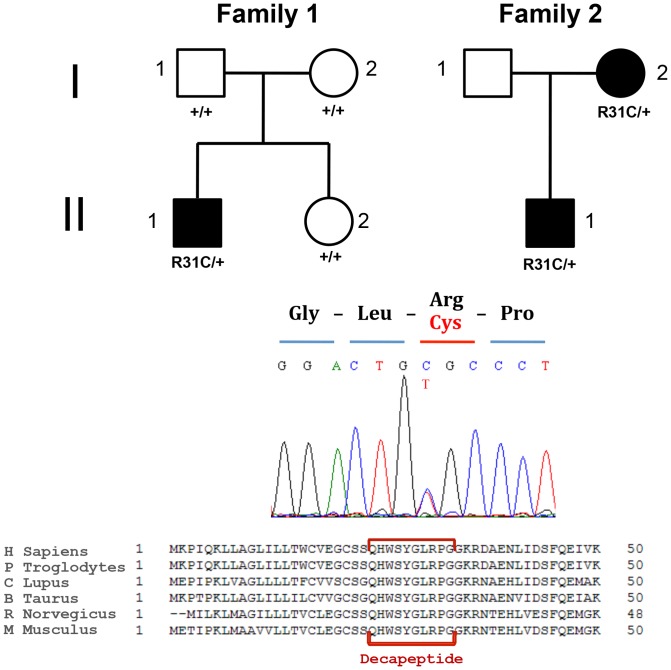
Pedigree of nCHH families carrying c.91C>T (p.R31[R, C]) *GNRH1* mutation. In the family 1, the *propositus* (II.1) has a *de novo* mutation and his filiation has been confirmed by DNA microsatellites. In the family 2 the mutation was transmitted from the mother (I.2) to her son (II.1). She required medical assistance for procreation. Clinical and demographic data of all patients are reported in Table 1. Electropherogram represents the heterozygous mutation in the individual II.1 from family 1. In the panel below, pre-pro-GnRH amino acid sequence conservation. Decapeptide is shown in red.

We identified the *GNRH1* c.91C>T (p.R31C) mutation in two unrelated French families with nCHH. Demographic, clinical, biological and genetic data are reported in Table 1. Interestingly, the two pedigrees are very different in terms of presentation (Fig. 1). In family 1, nCHH is sporadic. The boy (II.1) presented at 19 years old with a failure to progress through puberty. At physical examination he had a partial pubertal development with 12-mL bilateral testes volume. He had no olfactory impairment. Hormone assays revealed very low testosterone levels and low gonadotropin levels. No secondary causes were found for central hypogonadism (see Table 1). *GNRH1* heterozygous mutation in this boy was *de novo*, as ascertained by micro-satellites analysis.

**Table 1:** Clinical, biological and genetic characterization of patients.

	II.1 (Family 1)	II.1 (Family 2)	I.2 (Family 2)
Clinical data			
Sex	M	M	F
Age at diagnosis	19	21	65
Total testosterone (ng/mL) *	0.8	0.77	NA
Estradiol (pg/mL) *	NA	7	<12
LH (mU/mL) *	1	1	2.8
FSH (mU/mL) *	0,9	1.4	5.8
LH pulsatility	NA	Absent	NA
AMH (mmol/l) *	NA	1.4	<0.4
Inhibin B (ng/mL) *	NA	101	<15
Ferritin (mcg/mL)	63	56	103
Testicular volume (mL)	12	10	-
Ovarian volume (mL)	-	-	<10
Pituitary and olfactory IRM	Normal	3-mm Rathke's cyst	Normal
Olfactometry**	Normal	Normal	Normal
Associated features	None	None	None
Genetic data			
* GNRHR1*	normal	normal	normal
* KISS1*	normal	normal	normal
* GPR54*	normal	normal	normal
* PROK2*	normal	normal	normal
* PROKR2*	normal	normal	normal
* FGFR1*	normal	normal	normal
* FGF8*	normal	NA	NA
* NELF*	NA	normal	normal
* TAC3*	normal	normal	normal
* TACR3*	normal	normal	normal
* GNRH1 promoter*	normal	normal	normal
* GNRH1 exon 1*	normal	normal	normal
* GNRH1 intron 1*	normal	normal	normal

NA: not available.

* Reference ranges for hormone levels and method characteristics. Total testosterone: 3.5–8.5 ng/mL for adult men (RIA, Orion Diagnostica device, Spectria®, Espoo, Finland; detection limit: 0.01 ng/mL; intra- and interassay coefficients of variation (CVs): 3.2% and 4.6%); estradiol: 15–35 pg/mL for men, <16 for post-menopausal women (RIA, Orion Diagnostica, Spectria®; detection limit: 2 pg/mL; intra- and the interassay CVs: 2.8% and 5.8%); LH: 1.4–8 mU/mL for men, >30 mU/mL for post-menopausal women; FSH: 1.4–10 mU/mL for men, >30 mU/mL for post-menopausal women (for LH and FSH: Immunotech device, Beckman Coulter, Praha, Czech Republic; detection limits: 0.1 IU/L for both FSH and LH; intra- and interassay CVs: <6.3% for FSH, <6.7% for LH); AMH: 22–38 pmol/L for men, 14–48 for women (enzyme immunometric assay, Immunotech reagents, Beckman Coulter Company, Marseille, France; detection limit: 0.4 pmol/L; intra and interassay CVs: <12.3% and <14.2%,); Inhibin B: 80–327 pg/mL for men, <20 pg/mL for post-menopausal women (enzyme immunometric assay, Oxford Bio-Innovation reagents, Serotec, Oxford, UK; detection limit: 15 pg/mL; intra- and interassay CVs: 4.2% and 10.2%,).

** Subjective olfactometry was performed by a computed-assisted validated test [35].

In the second family the *GNRH1* mutation was present in heterozygosis and segregated with disease. The boy (II.1) was diagnosed having hypogonadotropic hypogonadism because of small testis volume (10 mL at left, 12 mL at right testis), low serum testosterone and low gonadotropins. His pubertal stage was P3 according to Tanner. He had no anosmia. Common causes of secondary hypogonadism were excluded. His mother (I.2) was affected by primary amenorrhea, and conceived after ovarian stimulation by exogenous gonadotropins, although a formal diagnosis was not established at that time. She was re-evaluated later at the age of 65, and hormone assays revealed low sex steroids accompanied with inappropriately low gonadotropins. She had no other apparent secondary causes (see Table 1). The father (I.1) was not available for genetic analysis.

We did not identify a second genetic event after genomic regulatory region analysis of *GNRH1* locus and *GNRH1* cDNA sequencing in all *propositii*.

### Predictive analysis

The *GNRH1* c.91C>T nucleotide substitution did not create any donor or acceptor aberrant splice site according to prediction tools with Alamut® software. This nucleotidic substitution induces a missense at codon 31, replacing Arginine-31 by a Cysteine in the pre-pro-GnRH (*GNRH1* p.R31C).

Prop v.1.0b predicted that this missense did not abolish proconvertase-dependent cleavage site for pre-pro-GnRH maturation into the decapeptide (amino-acids 24 to 33).

Alignment with orthologs revealed conservation of arginine in position 8 of GnRH in mammals (Fig. 1). This amino acid exchange was classified as deleterious by various *in silico* prediction tools (see methods section). More importantly, experiments on the substitution of arginine 8 with a variety of amino acids demonstrate that it is crucial for binding and signaling [10]. Arginine 8 was further shown by mutagenesis studies in the GnRH-R that it interacted with an acidic residue in the extracellular loop three of the mouse (glutamate 301) and the human (aspartate 302) GnRH-Rs [8], [9].

### In vitro molecular characterization

R31C and wild-type (WT) decapeptides were stable over 24-hours at room temperature in water and culture medium. No dimerization of the mutant decapeptide was found (Fig. S1).

**Figure 2:**
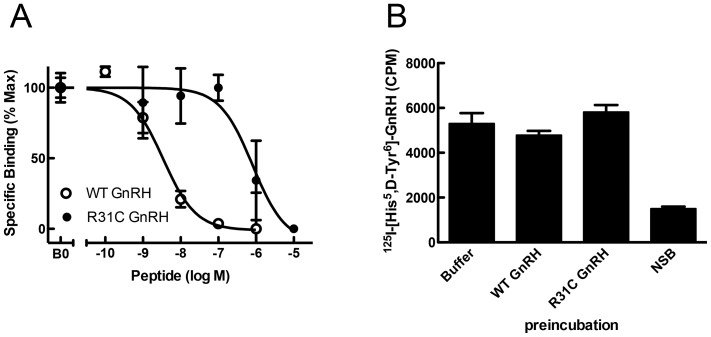
Binding of WT and R31C GnRH to the GnRH receptor (GnRH-R). (**A**) COS-7 cells were transiently transfected with a human GnRH-R DNA construct and incubated with a ^125^I-labeled GnRH agonist in the presence of indicated concentrations of WT GnRH or R31C GnRH. IC-50 values are WT GnRH, 2.8×10^−9^ M and R31C GnRH, 8.50×10^−7^ M. Data are given as means and SD of two experiments performed in duplicate. (**B**) Transfected COS-7 cells were pre-incubated with buffer alone, WT GnRH (10^−8^ M) or R31C GnRH (10^−5^ M) and then washed to allow dissociation of ligand not covalently attached to the GnRH-R. Pre-incubated cells were then incubated with ^125^I-labeled GnRH agonist in the absence or presence of saturating concentration of unlabeled WT GnRH (NSB). Data are the means of two experiments performed in triplicate.

**Figure 3:**
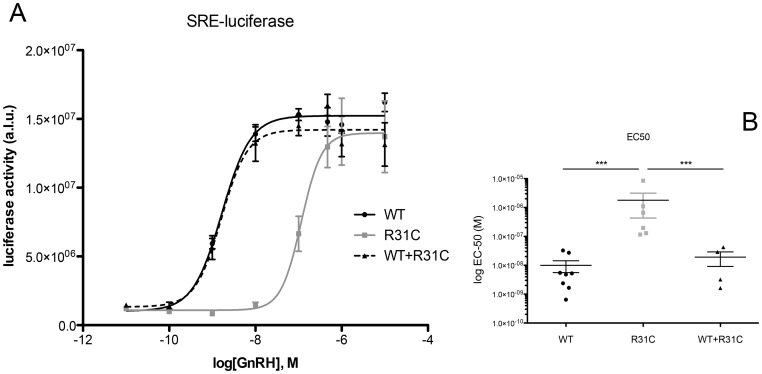
SRE-coupled luciferase assay. (**A**) HEK293T cells were exposed to graded concentrations of wild type (WT) GnRH, R31C and equimolar concentrations of WT + R31C during five hours. Luciferase activity arbitrary units by luminometry (a.u.) have been shown as ratio on beta-galactosidase activity by optical density used as transfection efficiency internal control. This experience was conducted eight times. (**B**) Representation of individual values of EC-50 (calculated with Arcsin “P” root transformation) from n = 8 dose-response experiments. WT GnRH EC-50 was calculated at 4±1.2 nM, R31C EC-50 at 313±130 nM. ***p<0.0001.

**Figure 4:**
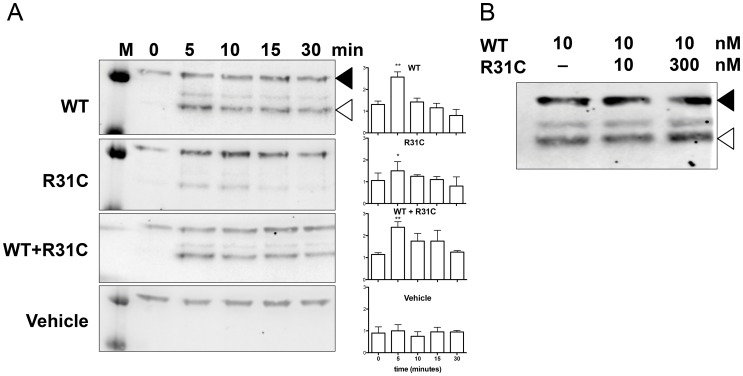
ERK1/2 phosphorylation by Western blot. (**A**) LbetaT2 cells were starved overnight in serum-free DMEM and then treated by 10 nM WT, 10 nM R31C, 10 nM WT +10 nM R31C peptides or vehicle for 0, 5, 10 and 30 minutes. Representative of four experiments. Density quantification is displayed on right-hand, and data are expressed as mean ± SD for four experiments. *p<0.05; **p<0.01 over baseline. (**B**) LbetaT2 cells were starved overnight in serum-free DMEM and then treated by a constant dose of WT GnRH (10 nM) and increasing (0–10–300 nM) doses of mutant R31C peptide after 5 minutes exposition. Lysates were analyzed by Western Blotting using phospho-ERK1/2 antibodies (Cell Signaling®, open arrow) and mouse monoclonal anti alpha-tubulin (Sigma Aldrich®, St. Louis, MO, close arrow). Similar results on ERK intensity and kinetics were obtained using transiently transfected HEK293T cells.

The R31C GnRH peptide bound the GnRH-R with an affinity more than 100-fold lower than that of WT GnRH (Fig. 2A and 2B). Dose-response curves with SRE-luciferase assay showed an almost 100-fold reduction of R31C agonist *versus* WT (Fig. 3A). The EC-50 was 4 nM for WT GnRH and 314 nM for R31C GnRH (p<0.0001, Fig. 3B). ERK1/2 phosphorylation was maximal at 5 minutes. R31C GnRH showed similar kinetics, but the degree of phosphorylation was reduced (Fig. 4A).

**Figure 5:**
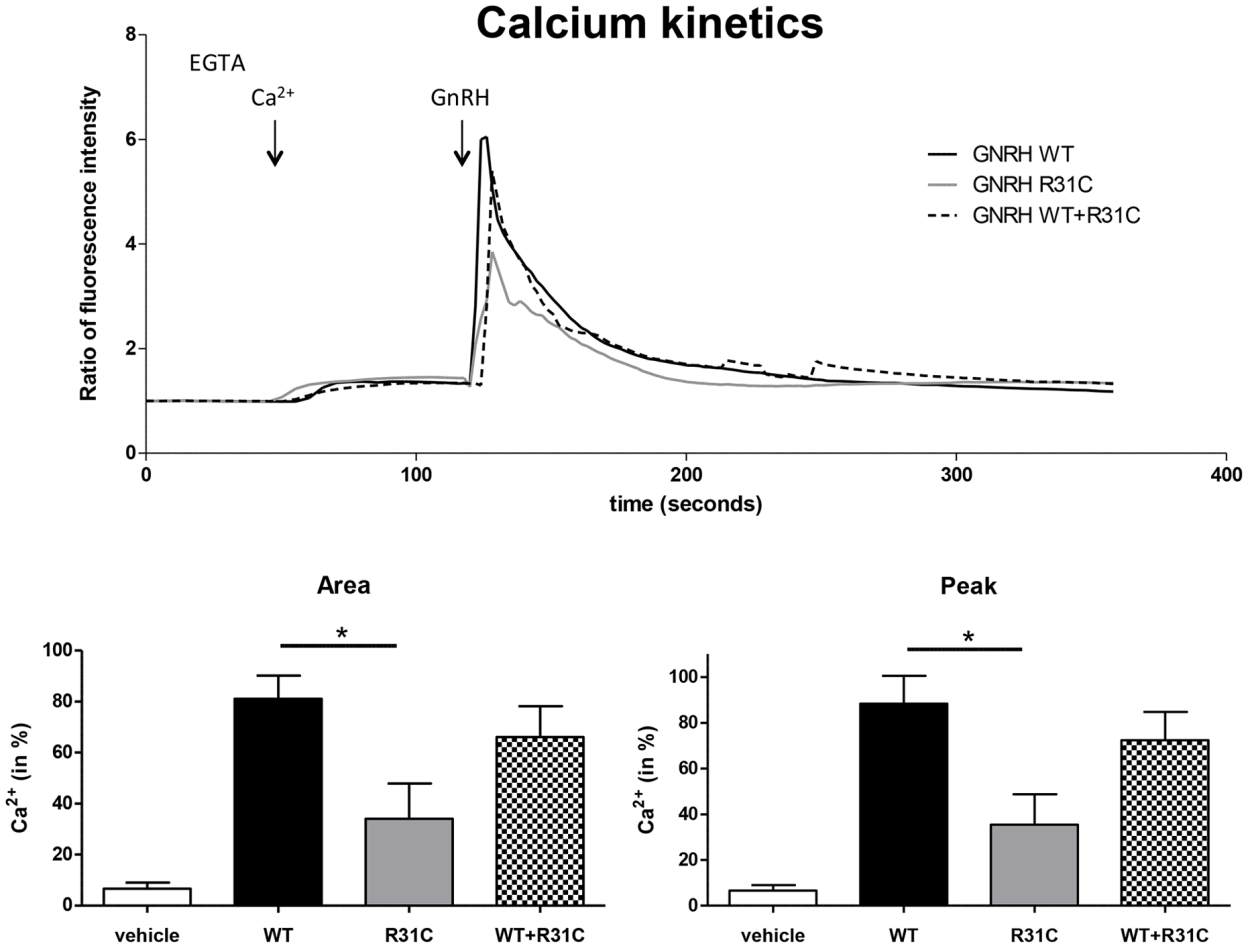
Effect of treatments on intracellular calcium mobilization. Cells were loaded with Fura 2-AM. At the time of the experiment 100 µM EGTA was added 30 sec before addition of 300 µM of CaCl_2_ (Ca^2+^) to the medium as indicated, and cells were then stimulated with 10 nM of wild type (WT), mutant (R31C) or equimolar combination (10 nM WT +10 nM R31C) or vehicle for 3 min. In the upper panel, representative curves of single cell recording. Each experiment mean was analysed using One-way ANOVA followed by Tukey's Multiple comparison test. In the lower panels, histograms show the means ± SEM (n = 4) of the response to ligands as calcium peak and as area under the curve (AUC). R31C-induced peak and AUC were significantly lower than WT. Combined treatment by WT+R31C did not induce a different calcium peak and AUC compared to WT alone. Representative of at least three independent experiments, *p<0.05.

**Figure 6:**
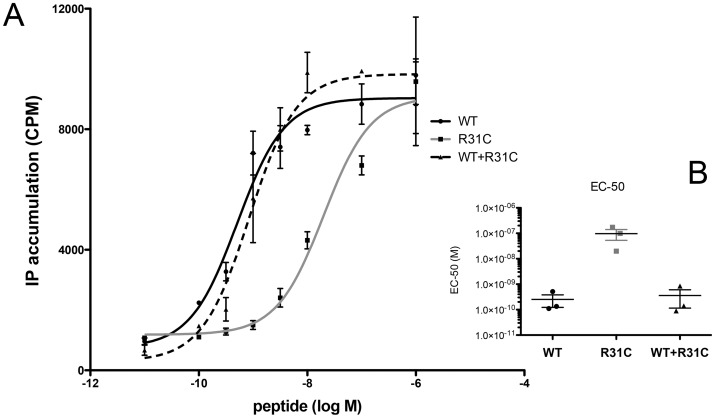
IP accumulation. (**A**) COS-7 cells were transiently transfected with human GnRH-R expression vector and washed before the addition of myo-[2-^3^H]Inositol. After 16 hours cells were washed and incubated for 1 hour with graded concentrations of WT, R31C and equimolar WT+R31C GnRH. This experience was conducted three times each in triplicate. (**B**) Representation of individual values of EC-50 (calculated with Arcsin “P” root transformation) from n = 3 dose-response experiments. WT GnRH EC-50 was calculated at 0.52±0.22 nM, R31C EC-50 at 198.8±75.9 nM, WT+R31C EC-50 at 0.63±0.42 nM.

WT GnRH induced a rapid and transient calcium mobilisation in LbetaT2 cells. Calcium response by R31C was significantly reduced compared to WT in terms of peak and area under the curve (p<0.05, see Fig. 5). Pre-treatment with 100 nM GnRH-antagonist cetrorelix abolished responses to both ligands (data not shown). The IP accumulation dose-response curve demonstrated that the R31C GnRH (EC-50, 199 nM) was more than a 100-fold less potent than WT GnRH (0.5 nM), (p<0.01, Fig. 6A and B). The maximal IP generated by both ligands was the same indicating that the R31C GnRH peptide was a full agonist, as was found in the SRE-luciferase assay.

**Figure 7:**
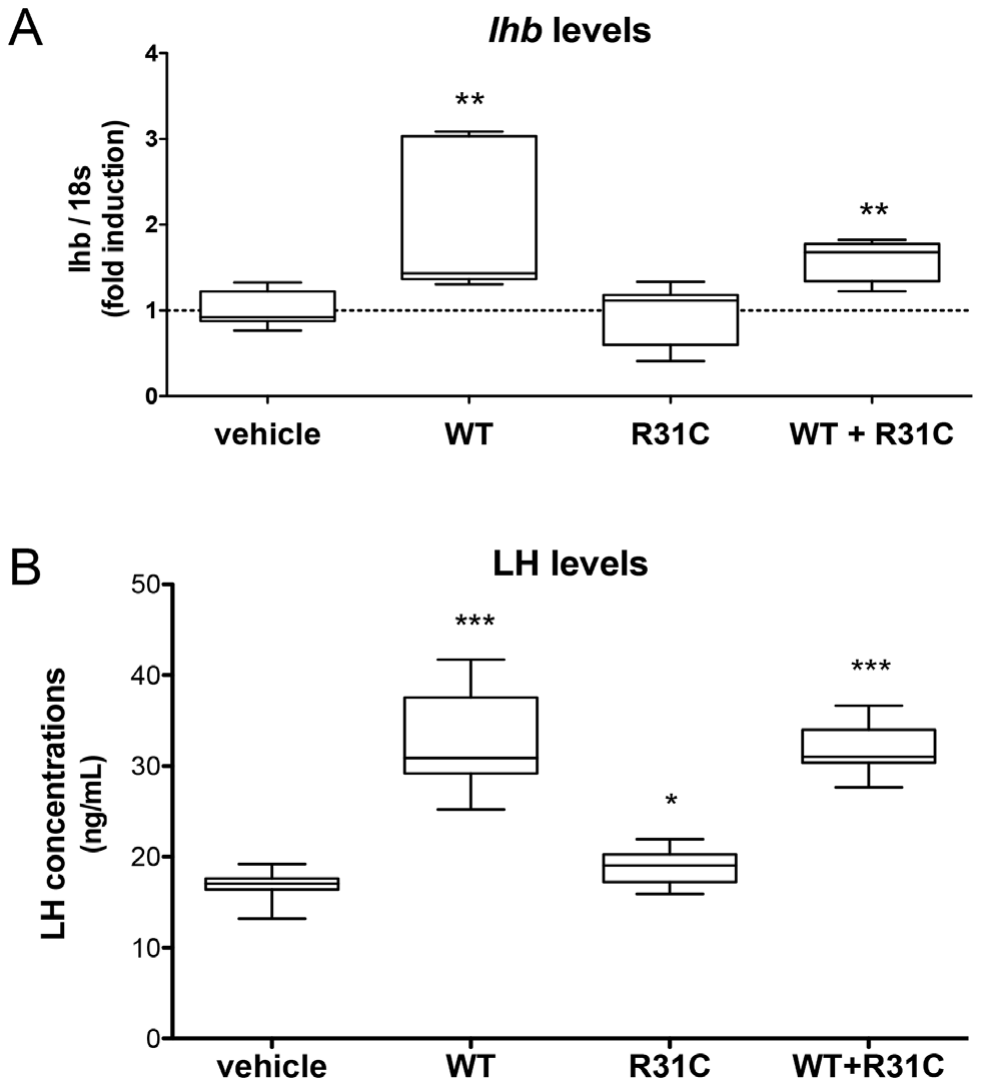
Effects of treatments on *lhb* transcription and LH secretion. (**A**) LbetaT2 cells were starved overnight in serum-free DMEM and treated either by 10 nM WT, R31C, 10 nM WT +10 nM R31C peptides or vehicle. After five hours treatment cells were lysed for RNA extraction (Trizol®). Mouse *lhb* levels were analyzed by quantitative real time RT-qPCR. Values are expressed as individual ratios on 18S and represented as fold induction on vehicle as means ± SEM, **p<0.01. This experience was conducted n = 7 times in duplicates. (**B**) LH concentrations from LbetaT2 cells culture *media*. Cells were starved overnight in serum-free DMEM and incubated with 10 nM WT, 10 nM R31C, 10 nM WT +10 nM R31C, or vehicle for 5 hours. LH levels were measured by a combined rat/mouse RIA. Values are expressed as individual values and as means ± SEM, *p<0.05, ***p<0.001. The experiment was conducted four times and each sample was assayed in duplicates.

WT GnRH significantly increased *lhb* transcript levels in gonadotropes (p<0.01), whereas R31C GnRH did not significantly increase these levels over baseline (not significant, Fig. 7A).

WT GnRH strongly stimulated LH secretion over baseline (p<0.001). R31C GnRH significantly stimulated LH secretion in LbetaT2 cells supernatants over baseline (p = 0.042, Fig. 7B).

The above findings clearly show that in binding, signaling and functional assays, the R31C GnRH is less potent than WT GnRH. Since the heterozygous condition was associated with nCHH in our patients, that could suggest a dominant negative effect, we studied the effects of combining WT GnRH with R31C GnRH.

We hypothesized that the cysteine in position 8 of the R31C GnRH might form disulfide bridges with cysteine residues in the receptor that are not in disulfide bridges. There are three such cysteines: cysteine 218, cysteine 279 and cysteine 317. In order to examine this possibility the effects of R31C GnRH pre-incubation on subsequent binding of a radio-labelled GnRH analog was examined. We determined from the competition binding study that R31C GnRH would occupy 50% of receptors. As a control, we utilized WT GnRH at 10^−8^ M, which also occupies 50% of receptors. After treatment, the cells were extensively washed to remove all non-covalently bound peptide. Subsequent binding of radiolabeled GnRH analog was the same in these treatments as well as in medium-treated cells (Fig. 2B). These findings indicate that both WT GnRH and R31C GnRH can be fully removed from the receptor, indicating that R31C GnRH does not form covalent disulfide bonds with the receptor.

Dose-response curves generated from SRE-coupled luciferase were indistinguishable from WT GnRH administered alone (Fig. 3A).

After pulsatile administration of each ligand at 90-minute frequency, WT GnRH was more potent than R31C GnRH to induce luciferase activity coupled to SRE reporter gene. Combination of WT and R31C was not able to impair luciferase activity when compared to WT alone (Fig. S2).

Treatment of LbetaT2 and GnRH-R-expressing HEK293T cells with 10 nM WT GnRH in combination with 10 nM R31C GnRH induced similar kinetics of ERK1/2 phosphorylation compared to 10 nM WT alone (Fig. 4A). Increasing the dose of R31C GnRH from 10 nM to 300 nM in the presence of 10 nM WT GnRH did not reduce ERK1/2 phosphorylation at 5 minutes exposure (Fig. 4B). Calcium peak and area under the curve after treatment with the combination of 10 nM GnRH and 10 nM mutant peptide was indistinguishable from WT GnRH alone (Fig. 5). IP dose-response curve with equal concentrations of WT and R31C peptides was indistinguishable from WT GnRH alone (Fig. 6). This indicates that a full range of doses of R31C GnRH does not influence the ability of WT to bind and activate the GnRH-R. R31C GnRH was also unable to influence the stimulation of *lhb* transcription (Fig. 7A) or LH secretion (Fig. 7B) from LbetaT2 cells by WT GnRH.

## Discussion

*GNRH1* mutations are a very rare cause of nCHH [5]–[7], [11], [12]. The *GNRH1* p.R31C mutation is the only description of an amino acid change in GnRH. Yet this mutation has been described in nine individuals in four separate families. This mutation was first reported in association with nCHH in heterozygosis and a dominant inheritance was postulated [6], contrasting with the previously described recessive transmission of the frame shift mutation, which fails to transcribe the GnRH sequence [5]. Some nCHH individuals with the R31C mutation concomitantly had variations in other nCHH genes perhaps suggesting oligogenism. More recently, the R31C mutation was reported in a girl with no variations in other nCHH and Kallmann Syndrome-related genes [7]. This is of interest since this study analyzed all known genes associated with nCHH and Kallmann Syndrome aiming to establish the prevalence of oligogenism in these syndromes. This mutation was considered not sufficient to explain the phenotype because of the heterozygous inheritance [7]. In the present study, we have found the same mutation in three additional nCHH patients from two unrelated families and unrelated to the previously described families with the R31C mutation. Although sex steroid and gonadotropin levels undoubtedly related to CHH, more-than 4 mL testicular volume in patients at presentation suggested a residual functional activity of hypothalamic-pituitary-gonadal axis, probably reflecting heterozygosity.

The identification of this recurrent mutation in four unrelated families, the finding of a *de novo* event in one family and the location of the nucleotide base change within a CpG islet are three consistent arguments for a mutational «hot spot». As interest, this amino acid is the most variable among vertebrate and invertebrate GnRHs [1]–[4]. The R31C GnRH mutation has never been identified in controls and segregation with the phenotype has been found in all affected families (see Table 2). Furthermore, this nucleotide base change has not been identified in 5989 individuals from the Exome Sequencing Project (ESP) cohort (NHLBI GO, Seattle, WA; URL: http://evs.gs.washington.edu/EVS; October, 2012). Thus, this implies that this missense mutation is likely responsible for the nCHH phenotype. However, although the R31C GnRH had very low activity in receptor binding, SRE-luciferase, IP, Ca^2+^, ERK1/2 signaling and in *lhb* gene expression and LH secretion, we were unable to demonstrate any dominant negative effect on WT GnRH activities.

**Table 2:** Recurrence of c.91C>T (p.R31C) *GNRH1* base change in subjects from CHH families and in healthy controls.

Authors	Reference	Year	CHH	Healthy individuals
Vagenakis et al.	[11]	2005	0/26	NA
Chan et al.	[6]	2009	4/310	0/192
Topaloglu et al.	[36]	2009	0/50	0/100
Quaynor SD. et al.	[7]	2011	2/48	0/188
Current study		2013	3/410^b^	0/545^b^
**Total**			9/844***	**0/1025**

NA: not applicable; *** p<0.0001 by Fisher's test between CHH patients and healthy controls. ^a^: the mother of the female *propositus* has been represented as a carrier but no clinical data are reported. ^b^: 120 CHH patients and 345 healthy subjects have been added to previously published data [5].

Previous genetic studies established that *GNRH1* loss of function mutations lead to an autosomal recessive nCHH. Thus we first searched for another genetic event at *GNRH1* locus. This was excluded by an exhaustive analysis of the entire sequence of intron 1 present in the hypothalamic primary transcript, the sequence of the upstream and downstream *GNRH1* promoters and the cDNA coding regions [13], [14]. We also ruled out oligogenism by sequencing the main genes associated with nCHH and Kallmann Syndrome [15]–[17], although *WDR11*, *CHD7* and *SEMA3A*, other genes known to be associated with CHH, have not been analyzed. Furthermore, we cannot entirely exclude the contribution of a gene not known to be associated with CHH. Next generation sequencing methods could be helpful to find a possible second genetic defect.

In order to investigate the possible mechanisms whereby R31C GnRH might influence the actions of WT GnRH, as it is apparently the case in the patients, we set about first examining the activity of the R31C GnRH in receptor binding and variety of signaling pathways, as well as its ability to stimulate LH release. We then examined its ability to affect WT GnRH actions in these systems. R31C GnRH had a binding affinity of more than 100-fold lower than that of WT GnRH. Consistent with this observation, R31C GnRH had similar reductions in potency (increased EC-50 values) in stimulation of SRE-luciferase and IP generation. However, R31C GnRH was able to elicit the same maximal stimulation of SRE-luciferase and IP, clearly demonstrating that it is a full agonist in recruiting these signaling pathways. This indicates that it has no antagonistic activity and it is therefore unlikely to explain the phenotype of the heterozygous patients. Supporting these observations, R31C peptide determined weaker responses in contrast to those of WT GnRH on ERK1/2 phosphorylation, Ca^2+^ mobilization, *lhb* transcription and LH secretion.

Further studies confirmed our interpretation that R31C GnRH does not antagonize WT GnRH actions at the receptor level. When R31C GnRH, at concentrations varying from 10^−10^ M to 10^−6^ M, was added in combination with WT GnRH at 10^−9^ M in various experiments, it failed to have any impact on receptor binding, SRE-luciferase, IP generation and Ca^2+^ signaling or on *lhb* transcription and LH secretion. These findings are in accordance with our understanding of structure-activity relations of GnRH analogs and their interaction with the GnRH-R. Arginine 8 is crucial for the correct conformation of mammalian GnRH and for its binding to the receptor [1]–[4]. In particular arginine 8 has been demonstrated to be crucial for the interaction with the aspartate 302 of the human GnRH-R and the glutamate 301 of the mouse GnRH-R [8], [9], [18].

The mode of inheritance, apparently dominant in four families, is thus not explained by a simple negative dominance in the parameters that we have measured. There are other possibilities that were not investigated here. Firstly, the mutant peptide may interact with molecules within the GnRH neuron to impair activity or induce toxicity, thereby reducing WT GnRH secretion. Aberrant transcription products might be retained in the endoplasmic reticulum or could act as neurotoxic agents, as already demonstrated for pro-dynorphin in ataxia [19] or arginine-vasopressin variants in diabetes insipidus [20]. Another possibility is that, in GnRH neurons, the R31C GnRH precursor forms an aberrant intermolecular disulfide bridge with the WT GnRH precursor, which disrupts correct folding of the molecule leading to detection by surveillance proteins and trafficking to lysosomes for degradation of both peptides. In addition, the heterodimer may be resistant to processing.

Although a wide range of cellular models was used, they do not necessarily reflect *in vivo* events. Despite abundant literature, the molecular mechanisms underlying GnRH-R down-regulation and pulse deciphering remain currently poorly understood [21]–[23]. It is of note that SRE-luciferase activity was not impaired after four pulses at 90-minute frequency (Fig. S2). Nevertheless a slow and progressive loss of pituitary response to mutant GnRH might require a longer pulsatile exposure. In this context, a transgenic mouse model may shed light on the mechanism of the dominant negative effect [24].

In conclusion, together with our report, four nCHH families carrying R31C *GNRH1* heterozygous mutations have been identified. As this is a putative «hot spot» mutation it is likely to be identified in other nCHH families. The families harboring the R31C mutation are of great interest for reproductive sciences since the pathophysiology is not explained by *in vitro* experiments. There is herein an obvious opportunity to study novel aspects of GnRH signaling *in vivo* and/or to identify novel genes modulating GnRH reproductive function.

## Materials and Methods

### Patients

From a cohort of 410 patients with congenital hypogonadism we screened for a panel of mutations including *GNRH1* (see below). The study was approved by the Paris Sud University Hospital ethics committee and complied with human research guidelines as stated in the Declaration of Helsinki. Patients gave their written informed consent before genetic analysis and hormone studies.

### Hormone assays

Serum LH, FSH, inhibin B, plasma testosterone and estradiol concentrations were measured by immunoradiometric, enzyme-linked immunoabsorbent, or radioimmuno-assays [25] as reported in Table 1.

### Genetic testing

Genomic DNA was isolated from white blood cells (WBC). Direct genomic sequencing of *GNRH1* was performed by sequencing all exons and exon-introns junctions (NG_016457.1), up-stream and down-stream promoter encompassing 1100 bp before start site of transcription (Fig. S3). Direct genomic sequencing of coding exons and intron-exon junctions of *GNRHR1*, *KISS1*, *GPR54*, *NELF*, *TAC3*, *TACR3*, *FGF8*, *FGFR1*, *PROK2* and *PROKR2* was performed as previously described [26]. PCR primers were designed by Primer Blast (http://www.ncbi.nlm.nih.gov/tools/primer-blast/) [26]. PCR and sequencing products were purified on a Biomek NXP-96 Laboratory Automation Workstation (Beckman Coulter, Villepinte, France) with Agencourt Ampure XP and Agencourt Cleanseq (Beckman Coulter Genomics, Danvers, MA). Sequencing products were analyzed with an automated capillary sequencer (ABI PRISM 3130xl Genetic Analyzer; Applied Biosystems, Foster City, CA). Electropherogram-derived sequences were compared with NCBI references using SeqScape Software 2.6 (Applied Biosystems, Foster City, CA).

Microsatellites genotyping was performed to confirm filiation (Powerplex 16 System®, Promega, Madison, WI).

Total RNA was extracted from cells (WBC or cells in culture) with the Trizol® reagent (InVitrogen, Cergy Pontoise, France). RT-PCR and direct cDNA sequencing was performed as previously reported [26], [27]. Complete CDS of *GNRH1* transcripts (NM_0001083111.1 and NM_000825.3) were analyzed with primers for RT-PCR and sequencing in Table S1.

### Molecular Characterization of the GnRH R31C mutant

#### Peptide custom and stability

“Wild type” (pGlu-His-Trp-Ser-Tyr-Gly-Leu-Arg-Pro-Gly-NH2) and “R31C” (pGlu-His-Trp-Ser-Tyr-Gly-Leu-Cys-Pro-Gly-NH2) mutant GnRH were synthesized by a custom peptide manufacturer (Eurogentec®, Liège, Belgium). Molecular weight was confirmed by MALDI-TOF and purity was assessed at ∼95% by the manufacturer.

Lyophilized products were suspended in sterile water in order to obtain 0.1 mM aliquots and conserved frozen at −150°C.

LC-MS/MS was performed with Quattro-LCZ triple quadrupole mass spectrometer equipped with the orthogonal electrospray source (Micromass, Manchester, UK) to analyze peptides stability in solution. Peptides solutions (water and cell culture medium) were incubated 24-h at room temperature for these stability studies.

*In silico* prediction of peptide cleavage was performed by means of Prop v.1.0b ProPeptide Cleavage Site (http://www.cbs.dtu.dk/services/ProP).

Prediction of protein function after selective amino acid substitution was obtained by means of Alamut® (Interactive Biosoftware, Rouen, France), AlignGVD (http://agvgd.iarc.fr/agvgd_input.php/), Polyphen2 (http://genetics.bwh.harvard.edu/pph2/), SIFT (http://sift.jcvi.org/) and PANTHER Coding SNP Analysis tool (http://www.pantherdb.org/tools/csnpScoreForm.jsp).

### Cell lines

Various cell lines were used for the experiments. HEK293T (ATCC CRL-11268) and COS-7 (ATCC CRL-1651), which do not express GnRH-R, were used after transient transfection with expression vector of human *GNRHR* (Missouri S&T cDNA Resource Center, Rolla, MO). Murine gonadotrope LbetaT2 cells were kindly provided by Dr. Mellon laboratory [28]. These cells endogenously express GnRH-R and are able to express gonadotropin subunit transcripts and to secrete mature LH glycoprotein after GnRH treatment [28].

### Competition radioligand binding assays

The high affinity GnRH analog, [His^5^, D-Tyr^6^]-GnRH was radio-iodinated as previously described [29] and purified using Sephadex chromatography [30]. COS-7 cells were transiently transfected with a human GnRH-R DNA construct containing the human GnRH-RII carboxy-terminal to enhance expression [31] using 6 µg of DNA and 30 µl FuGene HD (Promega Corporation, Madison, WI) per 10 cm dish and seeded into 12-well plates. Two days after transfection cells were washed with HEPES-DMEM containing 0.1% bovine serum albumin (2×1 ml, HEPES-DMEM-BSA) and incubated with ^125^I-[His^5^, D-Tyr^6^]-GnRH (100,000 CPM per well) and various concentrations of WT GnRH or R31C GnRH (4 h, 4°C) in a total volume of 0.5 ml. Cells were washed with phosphate-buffered saline (2×1 ml) and lysed with NaOH (1 ml, 0.1 M). Cell-bound radioactivity was counted in a gamma counter and IC-50 values were calculated using Graphpad Prism (GraphPad Software Inc, San Diego).

To determine whether R31C GnRH binds covalently to the GnRH-R, transfected COS-7 cells were incubated with WT GnRH (10^−8^ M), R31C GnRH (10^−5^ M) or HEPES-DMEM-BSA alone (2 h, 4°C), washed with HEPES-DMEM-BSA (1 ml) and incubated in HEPES-DMEM-BSA (5 ml, 1 h, 4°C) to allow dissociation of non-covalently bound peptide, before the binding assay was performed as above.

### Serum Responsive Element (SRE) luciferase assay

luc2P/SRE/Hygro plasmid (Promega, Madison, WI) was used to test luciferase production in response to MAP kinase activation as a reporter gene system. HEK293T cells (1.2×10^4^ cells/well) were seeded 72 h before testing in high-glucose Dulbecco's minimal essential medium (DMEM, Invitrogen, Cergy Pontoise, France) containing 2 mM glutamine, 100 IU/mL penicillin, 100 mg/mL streptomycin, and 10% heat-inactivated fetal calf serum at 37°C in 96-well plates. Twenty-four hours before testing, cells were co-transfected in serum free OptiMEM, using Lipofectamine 2000 (Invitrogen, Cergy Pontoise, France) with the plasmids for human GnRH receptor, luc2P/SRE/Hygro and pMIR-REPORT™ beta-galactosidase vector (Applied Biosystems, Foster City, CA). WT GnRH, R31C, WT+R31C or vehicle were added at different dilutions (from 10^−10^ to 10^−6^ M). After 5 h-incubation cells were harvested and assayed for luciferase activities as previously described [32], using a luminometer (Victor, Perkin Elmer, Waltham, MA). To standardize for transfection efficiency, the relative light units were normalized by the galactosidase activity at optical density. EC-50 and Emax values were calculated using Graphpad Prism (GraphPad Software Inc).

### Inositol phosphate (IP) accumulation

COS-7 cells were transiently transfected by electroporation with human GnRH-R DNA (10 µg/15 cm dish), seeded into 12-well plates and radiolabelled by overnight incubation with myo-[3H]Inositol (0.5 µCi/well, American Radiolabeled Chemicals, St Louis, Mo). Radiolabelled cells were washed and incubated (30 min, 37°C) in IP medium (HEPES-DMEM-BSA supplemented with 10 mM LiCl), then stimulated (60 min, 37°C) with various concentrations of WT GnRH or R31C GnRH or equal concentrations of WT GnRH and R31C GnRH. Incubations were stopped by removal of the medium and cells were lysed by addition of formic acid (1 ml, 10 mM). IP were extracted from cell lysates using Dowex 1 X8-200 chromatography and counted using a liquid scintillation counter (Pack ard). EC-50 and Emax values were calculated using Graphpad Prism (GraphPad Software Inc) [33].

### ERK1/2 Western blot

LbetaT2 cells were starved 18 h in serum-free DMEM before treatments, then exposed to 10 nM of WT, 10 nM of R31C, the combination of 10 nM of each peptide or vehicle. Cells were harvested at 0, 5, 10, 15 and 30 minutes after treatment. Western blotting analyses were performed as previously described [32].

### Measurement of intracellular free calcium concentration ([Ca^2+^]i)

LbetaT2 cells were kept in serum free medium overnight and then loaded with 2 µM Fura2-AM for 45 min at 37°C. The experiment was conducted in HEPES buffer (in mM; 116 NaCl, 5.6 KCl, 1.2 MgCl_2_, 5 NaHCO_3_, 1 NaH_2_PO_4_, 20 HEPES ph 7.4) in presence of extracellular Ca^2+^ (EGTA 100 µM + CaCl_2_ 300 µM). Single images of fluorescent emission at 510 nm under excitation at 340 and 380 nm were taken every 5 Sec. Changes in [Ca^2+^]i in response to GnRHs were monitored using the Fura2 340/380 fluorescence ratio. Basal ratio was arbitrarily considered as 1. Ca^2+^ responses over basal level were given either as maximal rise of [Ca^2+^]i or as area under the curve (for 2 min after agonist addition).

### Gene expression study

LbetaT2 cells were grown at 10^6^/well in 6-well plates and starved in serum-free DMEM 18 h before tests. After 5 hours incubation with 10 nM WT, R31C, the combination of 10 nM WT +10 nM R31C peptides or vehicle, cells were washed with 1× PBS and total RNA isolated using Trizol® (Invitrogen, Germany).

*Lhb* (murine LH beta subunit) transcript was quantified by real-time RT-PCR, using an ABI Step One Sequence Detector (Applied Biosystem, Foster City, CA) as previously described [27]. Primers are provided in Table S1.

### LH assay

LbetaT2 cells were starved overnight in serum-free DMEM. The test day cells were exposed to 10 nM WT, R31C, the combination of 10 nM of each peptide or vehicle during five hours. Cell culture supernatants were collected and rapidly stored at −80°C. In cell culture media, LH concentration was measured using a previously described ELISA method [34] with reagents supplied by Dr. Parlow (National Hormone and Peptide Program, Harbor-UCLA Medical Center, Torrance, CA). The minimum detectable LH concentration was 0.2 ng/ml, and the interassay coefficient of variation was less than 10%.

### Statistical analyses

Only nonparametric tests were used. Friedman's test was used to compare three or more matched groups and Kruskall-Wallis test for unmatched groups. These analyses were followed by Dunn's post comparison test. Differences were significant when p<0.05 (*p<0.05, **p<0.01, ***p<0.001). Statistical analyses were performed using GraphPad Prism version 5.0d (GraphPad Software Inc., San Diego, CA).

## Supporting Information

Figure S1 **Peptide stability on mass spectrometry-coupled electrospray (Quattro-LC).** Peptides were measured in aqueous solution at pH 7 and starved overnight at 37°C. WT and R31C decapeptides are found at the expected molecular weights (1181.6 and 1128.5, respectively). Formation of smaller fragments was absent in WT and negligible in R31C. Formation of R31C dimers was absent.
(PPT)

Figure S2 **Luciferase activity after pulsatile exposure.** HEK293T cells were transiently transfected with GNRHR and SRE-coupled luciferase reporter gene, and then exposed to four 90 min-spaced pulses of 10 nM WT, 10 nM R31C and 10+10 nM WT+R31C. After 5 minutes exposure to each ligand, cells were washed, and a subsequent pulse was given 90 minutes after. Five hours after the last pulse, cells were harvested for luciferase assay. Luciferase activity arbitrary units obtained by luminometry (a.l.u.) are shown as ratio on beta-galactosidase activity by optical density (used as transfection efficiency internal control). This experience was conducted three times (n = 8 replicates for each experiment).
(PPT)

Figure S3 **Genomic localization of human** ***GNRH1*** **and related transcription and translation products.** Two main regulatory regions are located upstream the trascription start site: the proximal promoter mainly regulates hypothalamic trascript, whereas the distal promoter controls a longer *GNRH1* transcript (retaining entire intron 1 sequence) in the extra-cerebral tissues. Amino acids are represented by letters from the international nomenclature. In the prepropeptide GnRH, functional domains are represented for the signal peptide (23 amino acids, blue), decapeptide GnRH (purple), and GnRH-associated peptide (GAP) (56 amino acids, green) (adapted from Bouligand et al., NEJM, 2009).
(PPT)

Table S1 **Primer sets used for experiments.**
(DOC)
