# Correction: R31C *GNRH1* Mutation and Congenital Hypogonadotropic Hypogonadism

**DOI:** 10.1371/annotation/1f10cc18-4cc4-49b1-9679-a1d16a1bb9fa

**Published:** 2013-08-21

**Authors:** Luigi Maione, Frederique Albarel, Philippe Bouchard, Megan Gallant, Colleen A. Flanagan, Regis Bobe, Joelle Cohen-Tannoudji, Rosario Pivonello, Annamaria Colao, Thierry Brue, Robert P. Millar, Marc Lombes, Jacques Young, Anne Guiochon-Mantel, Jerome Bouligand

A funding organization and two grants were incorrectly omitted from the Funding Statement. The first sentence of the Funding Statement should read: "This work was supported in part by grants from Paris-­Sud 11 University (Bonus Qualité Recherche 2009, Attractivité Univ. Paris Sud 2010), from PHRC HYPOPROTEO P081212, Fondation pour la Recherche Médicale and from Agence Nationale de la Recherche Genopath (ANR KALGENOPATH, ANR-09-GENO-017-01)." 

