# Supplementary figures and images for: R31C GNRH1 Mutation and Congenital Hypogonadotropic Hypogonadism
Source: PLoS One. 2013 Jul 25;8(7):e69616. doi: 10.1371/journal.pone.0069616 (PMC3723855; doi:10.1371/journal.pone.0069616)

## Slide 1
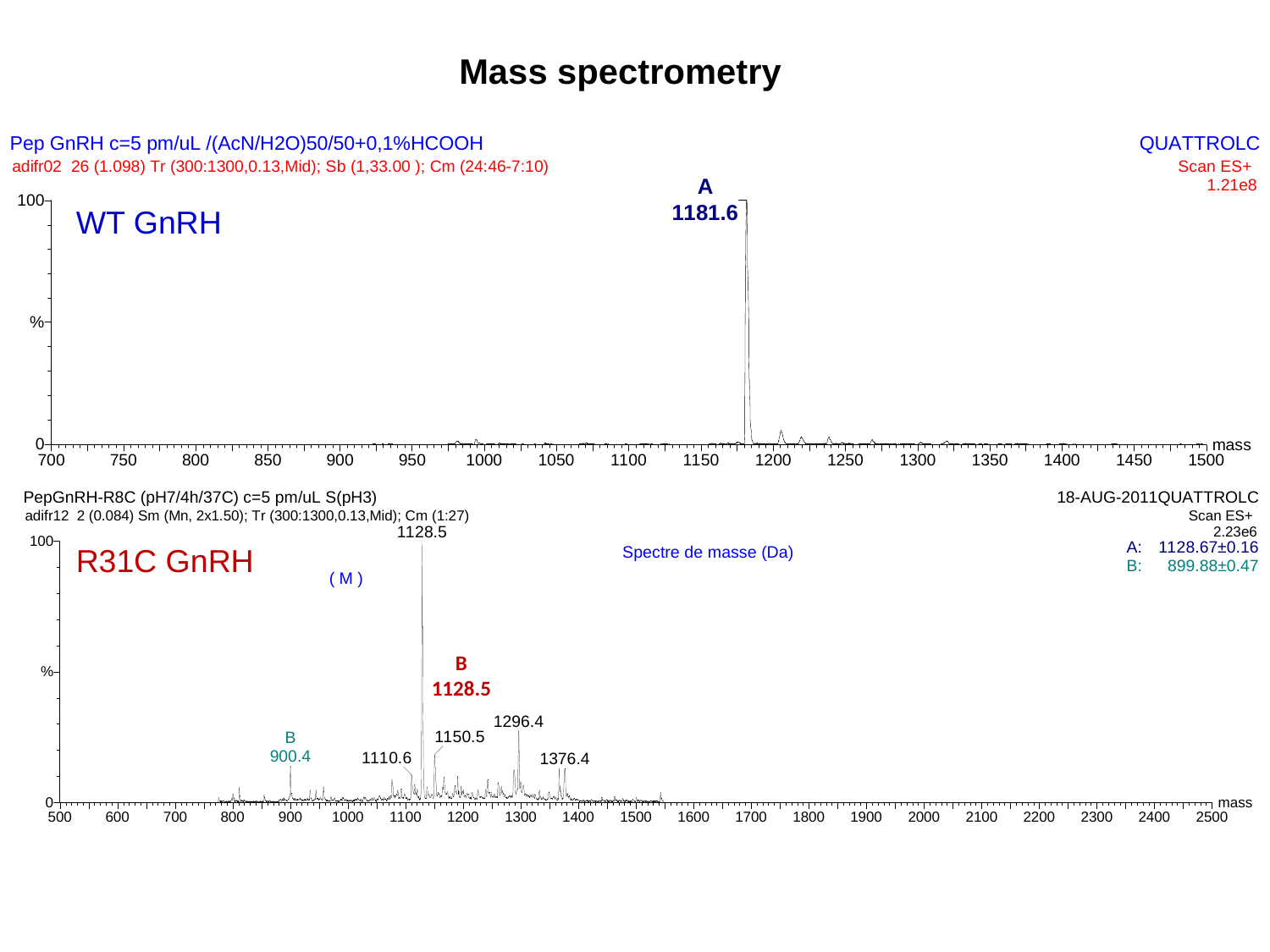

Mass spectrometry
WT GnRH
R31C GnRH
B
1128.5

Supplement: Figure S1 — Peptide stability on mass spectrometry-coupled electrospray (Quattro-LC). Peptides were measured in aqueous solution at pH 7 and starved overnight at 37°C. WT and R31C decapeptides are found at the expected molecular weights (1181.6 and 1128.5, respectively). Formation of smaller fragments was absent in WT and negligible in R31C. Formation of R31C dimers was absent. (PPT) [file pone.0069616.s001.ppt]

## Slide 1
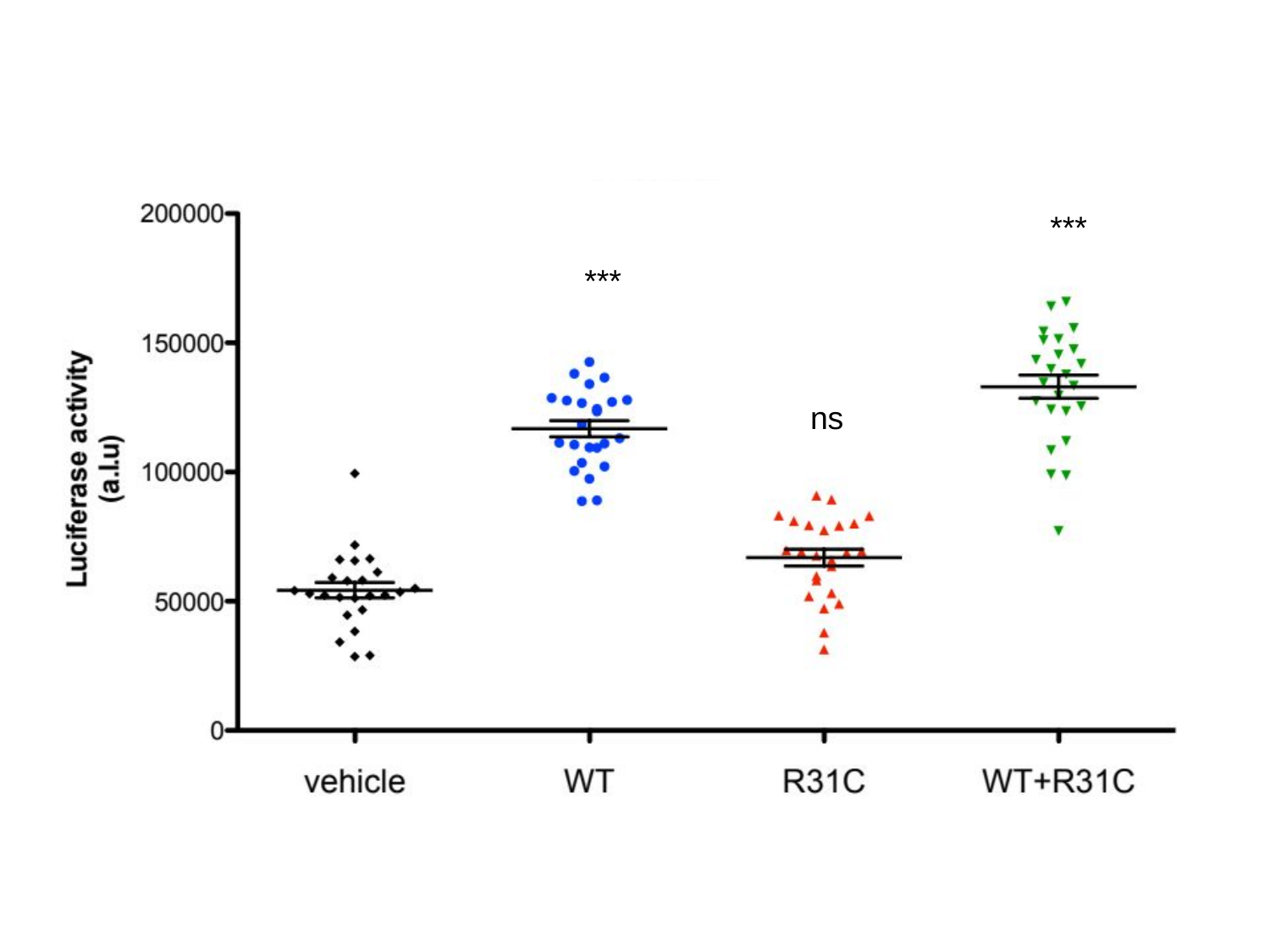

***
***
ns

Supplement: Figure S2 — Luciferase activity after pulsatile exposure. HEK293T cells were transiently transfected with GNRHR and SRE-coupled luciferase reporter gene, and then exposed to four 90 min-spaced pulses of 10 nM WT, 10 nM R31C and 10+10 nM WT+R31C. After 5 minutes exposure to each ligand, cells were washed, and a subsequent pulse was given 90 minutes after. Five hours after the last pulse, cells were harvested for luciferase assay. Luciferase activity arbitrary units obtained by luminometry (a.l.u.) are shown as ratio on beta-galactosidase activity by optical density (used as transfection efficiency internal control). This experience was conducted three times (n = 8 replicates for each experiment). (PPT) [file pone.0069616.s002.ppt]

## Slide 1
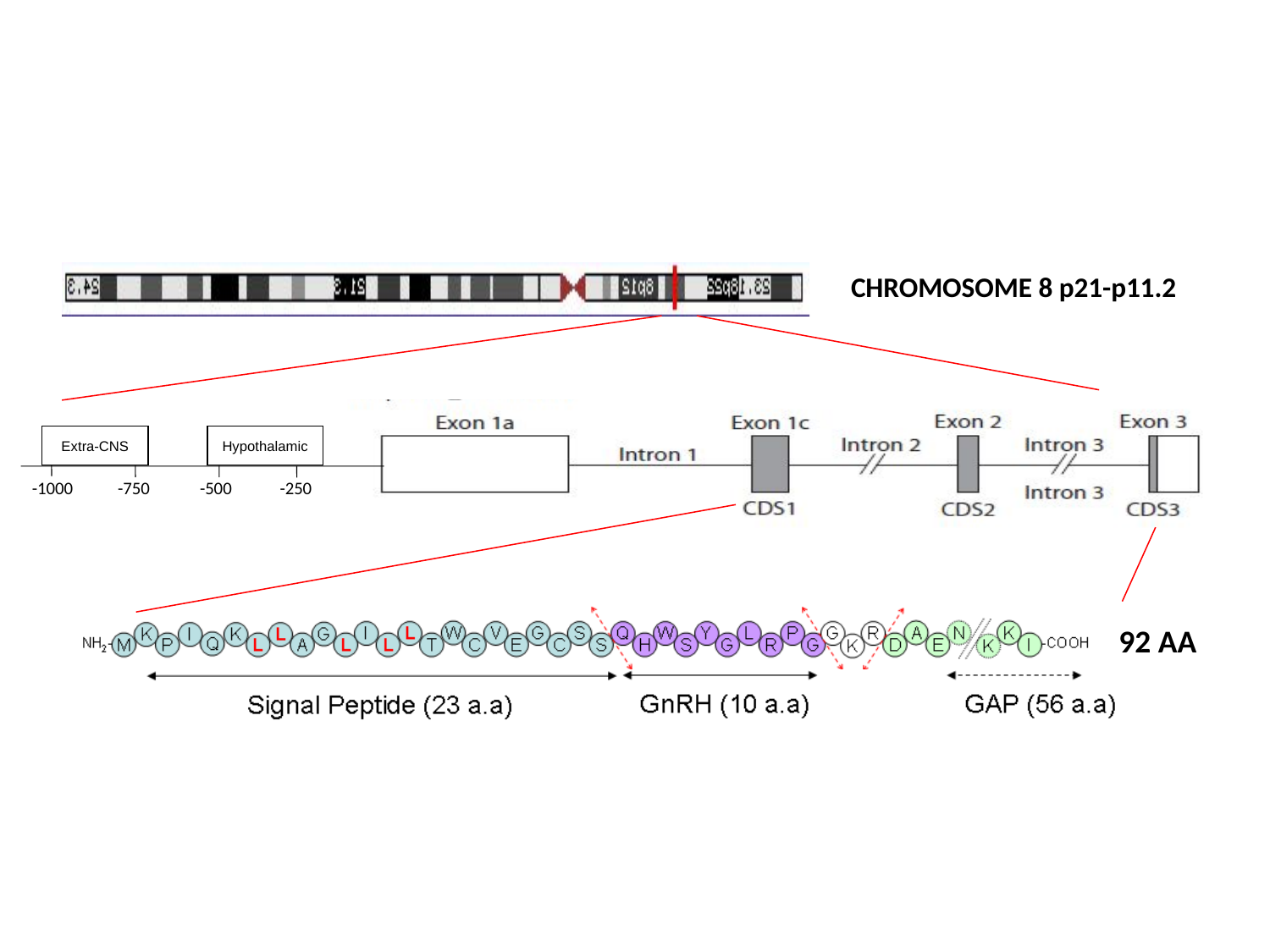

CHROMOSOME 8 p21-p11.2
Extra-CNS
Hypothalamic
-1000
-750
-500
-250
92 AA

Supplement: Figure S3 — Genomic localization of human GNRH1 and related transcription and translation products. Two main regulatory regions are located upstream the trascription start site: the proximal promoter mainly regulates hypothalamic trascript, whereas the distal promoter controls a longer GNRH1 transcript (retaining entire intron 1 sequence) in the extra-cerebral tissues. Amino acids are represented by letters from the international nomenclature. In the prepropeptide GnRH, functional domains are represented for the signal peptide (23 amino acids, blue), decapeptide GnRH (purple), and GnRH-associated peptide (GAP) (56 amino acids, green) (adapted from Bouligand et al., NEJM, 2009). (PPT) [file pone.0069616.s003.ppt]
