# Supplementary material for: R31C GNRH1 Mutation and Congenital Hypogonadotropic Hypogonadism
Source: PLoS One. 2013 Jul 25;8(7):e69616. doi: 10.1371/journal.pone.0069616 (PMC3723855; doi:10.1371/journal.pone.0069616)
Supplement: Table S1 — Primer sets used for experiments. (DOC) [file pone.0069616.s004.doc]

**Supplementary Table 1**

| **Genetic region** | **Forward** | **Reverse** |
| --- | --- | --- |
| **GNRH1 Promoter, distal** | TCCCAGCTGTAAAATGAACAAA | CACTCTTAAGGGCAGTGGGA |
| **GNRH1 Promoter, mid** | GGTTGAAAAACAGTCTGTCCCTA | CCAGGTAAGGCCTAATGCTG |
| **GNRH1 Promoter, proximal** | TGCTGTGTTCATCTTAATAATTTCA | AGTATAACTGCAAATAGAGGAGAAT |
| **GNRH1 Intron1, distal** | CCAGCAGGAAAGATTTCAATG | TGCTTGACATTACATGGCTACC |
| **GNRH1 Intron1, proximal** | ATGGTTCAGCCAGCAGTAATG | GGCATCTCTCTTTCCTCCAG |
| **GNRH1 exon1, 5’-UTR, distal** | ACCCACTTTGCATGGCTATT | TTGAGTCAGGATGTCTGGGA |
| **GNRH1 exon1, 5’-UTR, proximal** | GGATCCTACATGGACTTGGTATATA | TCCCAATCTTCCCTTGAAGT |
| **GNRH1 cDNA, part 1** | TCTTTCTGTTCCCACTGCCC | TGTAATTGGAACACCCCCTGG |
| **GNRH1 cDNA, part 2** | TGGCTCTCTGCCTCTAAACA | AGCCACTGGGGACAAAATGA |
| **GNRH1 cDNA, part 3** | CAAGCCAGCAAGTGTCTCTG | TGGAATATGTGCAACTTGGTGT |
| **Lhb** | TACTGTCCTAGCATGGTCCGAGTA | TGAGGGCTACAGGAAAGGAGACT |

5’-UTR: 5-prime untranslated region
